# Design of a High-Speed Digital System for Triple Discrimination Based on Stilbene-^6^Li Glass Composite Scintillators Detector

**DOI:** 10.3390/s26020690

**Published:** 2026-01-20

**Authors:** Qingyang Liu, Jiaqi Wang, Ye Chen, Zhiyuan Li, Zhenyu Wang, Hongzhao Zhou, Hengyi Su, Zungang Wang

**Affiliations:** 1 State Key Laboratory of Chemistry for NBC Hazards Protection, Beijing 102205, China; 2 CAEA Innovation Center of Nuclear Environmental Safety Technology, Southwest University of Science and Technology, Mianyang 621010, China

**Keywords:** composite scintillator, triple discrimination, sampling rate, high-speed digital, analog-to-digital converter (ADC), field-programmable gate array (FPGA)

## Abstract

This paper presents a design for a high-speed digital prototype system for discriminating fast neutrons, thermal neutrons, and γ-rays. The system uses a stilbene–^6^Li glass composite scintillator with excellent pulse shape discrimination (PSD) properties as the neutron detector. The PSD performance was investigated at different sampling rates, revealing stable performance at rates above 250 MSPS. The system core is a high-speed acquisition board based on the AD9434 analog-to-digital converter (ADC) and the ZYNQ7020 field-programmable gate array (FPGA), which acquires detector signals and implements real-time algorithms. The system was energy-calibrated with ^22^Na, ^137^Cs, and ^60^Co γ-ray sources and evaluated in a n–γ mixed field. Under an ^241^Am–Be neutron source, the system achieved Figure of Merit (FOM) values of 1.26 for fast neutron/γ, 2.18 for fast neutron/thermal neutron, and 3.25 for γ/thermal neutron discrimination above the 50 keVee electron equivalent energy threshold. These results are consistent with the analysis of down-sampled data from a DT-5730 digitizer, confirming that the system meets its design objectives. Additionally, the measured false alarm rates (FAR) were 0.33% for ^60^Co, 0.34% for ^137^Cs, and 0.26% for ^22^Na. This system integrates waveform discrimination and energy spectrum measurement capabilities, providing a high-performance, cost-effective electronic solution for high-speed signal acquisition and real-time processing in novel composite scintillator neutron detectors.

## 1. Introduction

In the fields of national security, nuclear non-proliferation, and fundamental research, detection technologies for fast and thermal neutrons have garnered considerable interest [1,2,3]. Conventional neutron detection methods rely primarily on ^3^He for thermal neutron detection, while employing moderators to capture fast neutrons [4]. Owing to the persistent scarcity and high cost of ^3^He resources, and the fact that the method can only be detected by completely slowing down fast neutrons to thermal neutrons, the development of detection technologies capable of efficiently detecting both thermal and fast neutrons has become an urgent priority.

Scintillator detectors are increasingly favored as alternative radiation sensors due to their high detection efficiency, substantial light yield, short decay time, strong radiation hardness, operational stability, and low cost. However, single scintillator materials are generally incapable of simultaneously detecting both thermal and fast neutrons. This limitation has prompted the development of composite scintillator detectors that integrate multiple sensitive materials.

Among organic scintillators, stilbene crystals have attracted considerable research interest for neutron detection [5,6,7,8] because of their superior pulse shape discrimination (PSD) performance and high detection efficiency for fast neutrons. When coupled with ^6^Li-glass, stilbene allows for the simultaneous detection and discrimination of fast neutrons, thermal neutrons, and gamma rays [9,10,11,12]. This composite design effectively mitigates the poor response of stilbene to thermal neutrons while compensating for the inherent limitation of lithium-glass, which is primarily sensitive to thermal neutrons. Furthermore, such composite scintillators offer a compact and lightweight design, demonstrating significant potential to replace traditional detection systems employing polyethylene moderators with ^3^He proportional counters. Consequently, they are emerging as a promising technology in the field of neutron detection.

PSD technology is fundamental to neutron detection using composite scintillators [13,14]. Over recent decades, progress in high-speed electronics and digital signal processing (DSP) has established digital PSD as a major research focus. This method involves sampling detector pulses with an analog-to-digital converter (ADC) and subsequently analyzing them using DSP algorithms, enabling operation at higher count rates. Currently, discrimination algorithms are primarily executed offline via software, where signal pulses are acquired by transient recorders or oscilloscopes for subsequent processing. However, this approach demands substantial data storage and cannot provide real-time discrimination in applications with high neutron and γ-ray fluxes [8,15,16,17,18].

This paper presents the design of a composite scintillator detector for a triple discrimination system based on a high-speed ADC and a high-performance field-programmable gate array (FPGA). The sampling rate of the ADC was determined by investigating the PSD performance at different sampling rates, achieving an optimal balance between system performance and cost-effectiveness. The system was energy-calibrated using three γ-ray sources, and its performance in triple discrimination and false alarm rate was tested the neutron filed. Finally, a comparative analysis with the CAEN DT-5730 digitizer and other reported systems was conducted, leading to a discussion on the relative merits of the proposed system and proposed directions for its further improvement.

## 2. Structure of Neutron Detector Based on Composite Scintillator

**Figure 1:**
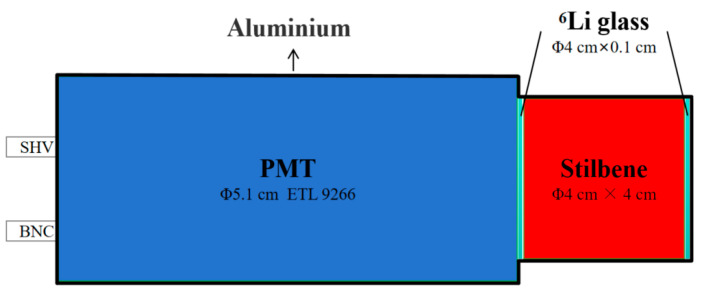
Schematic diagram of the composite scintillator detector structure.

A composite scintillator detector with a “sandwich-like” layered structure was designed for the simultaneous detection of fast neutrons, thermal neutrons, and γ-rays, as illustrated in Figure 1. The detector consists of a Φ4 cm × 4 cm stilbene crystal, optically coupled on both sides with Φ4 cm × 0.1 cm ^6^Li-enriched glass plates (activator: Ce, weight fraction of ^6^Li: 6.271%). One of the glass plates was coupled to an ETL 9266B (minimum wavelength: 290 nm) photomultiplier tube (PMT). The stilbene crystal, grown via the solution method, exhibits superior n/γ discrimination performance compared to EJ-276D plastic scintillators and EJ-309 liquid scintillators [7,11], and the lithium-glass had a lithium enrichment level of up to 95%. The key properties of the stilbene crystal and the ^6^Li glass are summarized in Table 1. Since the decay time of the ^6^Li glass is considerably longer than that of the stilbene crystal, it enables more effective discrimination of thermal neutron signals. Optical grease was applied to all coupling interfaces—between the stilbene, the ^6^Li glass, and the PMT—to minimize the loss of scintillation light. Meanwhile, all surfaces of the scintillator were wrapped with ESR reflective film except for the interface coupled with the PMT in order to maximize the collection efficiency and thus improve the detector performance and the quality of the output signal.

The fabricated composite scintillator detector, which integrates the scintillators, PMT, and voltage divider circuit, is housed within a light-tight enclosure constructed from aluminum and stainless steel. The base of the housing features connectors for high-voltage input and signal output.

## 3. PSD Performance with Different Sampling Rates

Although a higher sampling rate generally improves PSD performance, it simultaneously increases power consumption, memory usage, and system cost. Moreover, the excessively large data volume generated can significantly impede the speed of subsequent digital signal processing and analysis.

### 3.1. Mixed Pulse Collection

**Figure 2:**
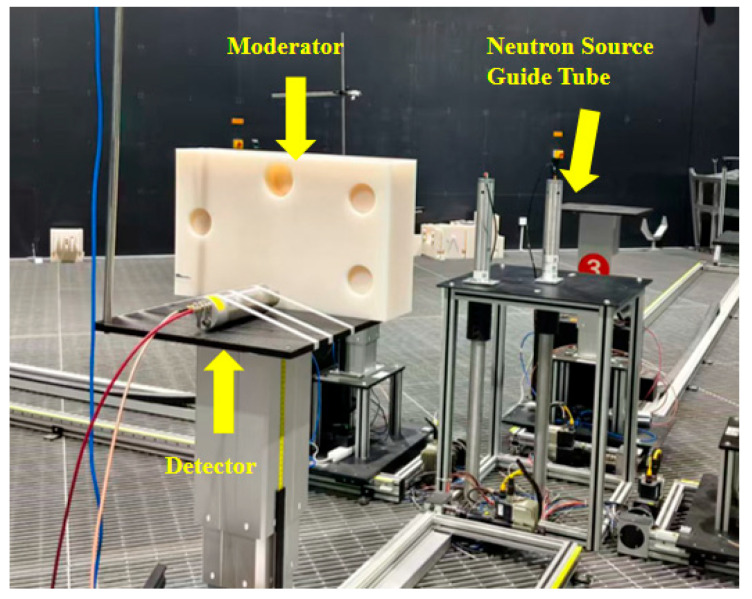
Mixed pulse signal acquisition experiment physical object.

Mixed pulse signals from the composite scintillator detector were acquired using a CAEN DT-5730 high-speed digitizer (500 MSPS sampling rate, 14-bit resolution) in a 20 Ci ^241^Am–Be neutron field. The neutron emission rate of the source was approximately 4.19 × 10^7^ s^−1^. The detector was positioned 1 m from the neutron guide source, with an 8 cm thick polyethylene moderator placed between them to enhance the thermal neutron flux. A laser collimator was used to ensure that the radiation from the source impinged perpendicularly on the detector. The digitizer was operated in DPP-PSD mode, with a dynamic range of 2 V_pp_ and a gain of 10 fC/(LSB × V_pp_). The pulse record length and pre-trigger time were set to 400 ns and 50 ns, respectively. Controlled by the CoMPASS_1.5.0 software, the digitizer acquired and stored 40,000 pulse signal events. The physical setup of the mixed pulse signal acquisition experiment is shown in Figure 2.

**Figure 3:**
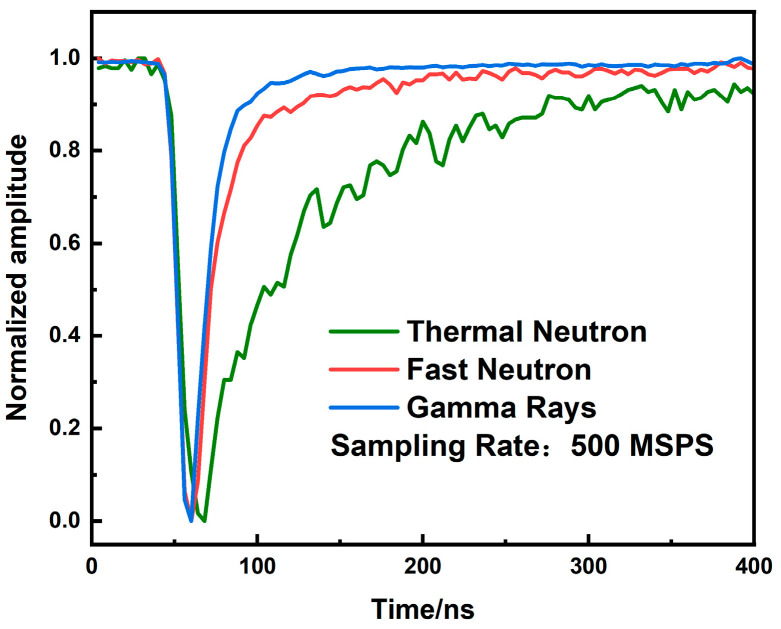
Three different particle waveforms from a composite scintillator detector.

Figure 3 presents the waveforms of three typical signals after baseline restoration and pulse amplitude normalization. The baseline was determined by averaging the first ten and last ten sampling points of each pulse signal, with the resulting average value then subtracted from all other sampling points. The detector outputs negative-polarity pulses, as shown in the figure. While all three pulses exhibit similar nanosecond-scale rise times, their fall times differ significantly. The γ-ray signal decays the fastest, followed by the fast neutron signal, with the thermal neutron signal displaying the slowest decay. These distinct fall times enable effective particle discrimination using PSD techniques.

### 3.2. The Method of PSD

The observed differences in the decay times of the acquired pulse waveforms provide a physical basis for discriminating among the different particle types. To quantify and discriminate these pulses, the Charge Comparison Method (CCM) is employed in the following analysis. The CCM discriminates between neutrons and γ-rays by utilizing the difference in the ratio of the slow-decay component to the total charge of the pulses generated by proton and electron recoils. Owing to its low computational cost, short processing time, and strong noise immunity compared to other methods, CCM is widely employed for n/γ discrimination in organic scintillators [20,21,22,23,24].

**Figure 4:**
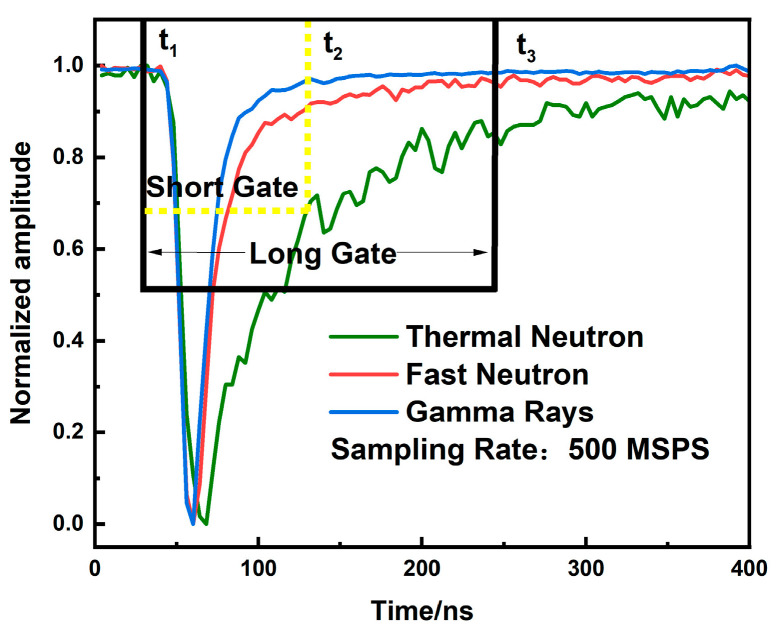
Schematic diagram of CCM.

The principle of CCM is illustrated in Figure 4. After determining the pulse peak position using Constant Fraction Discrimination (CFD)—a method where the original pulse is split into two paths: one inverted and the other attenuated by a factor of 0.75, with the sum of the two paths crossing zero at the reference time point—the start point t_1_ for both long and short gate integration is set at 50 ns before this reference, during the pulse pre-rise phase. Two additional points, t_2_ and t_3_, are selected on the falling edge and the tail of the pulse, respectively. The interval from t_1_ to t_2_ is defined as the short gate, and that from t_1_ to t_3_ as the long gate. The integrated charges within these gates are denoted as *Q_short_* and *Q_long_*, respectively. The Pulse Shape Parameter (PSP) is then calculated as follows:

(1) $$PSP=\frac{Q_{\mathrm{short}}}{Q_{\mathrm{long}}}$$

**Figure 5:**
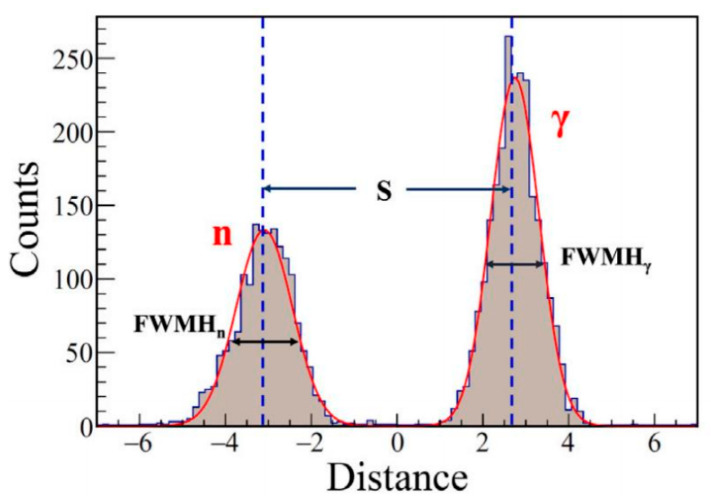
PSP count statistics distribution chart [26].

The Figure of Merit (FOM) in Equation (2) is used to evaluate the discrimination performance between different particles [25].

(2) $$FOM=\frac{S}{FWHM_{1}+FWHM_{2}}$$

where *S* is the distance between the peaks, *FWHM*_1_ and *FWHM*_2_ are full width half maximum of the two peaks, shown in Figure 5.

### 3.3. PSD Results

**Figure 6:**
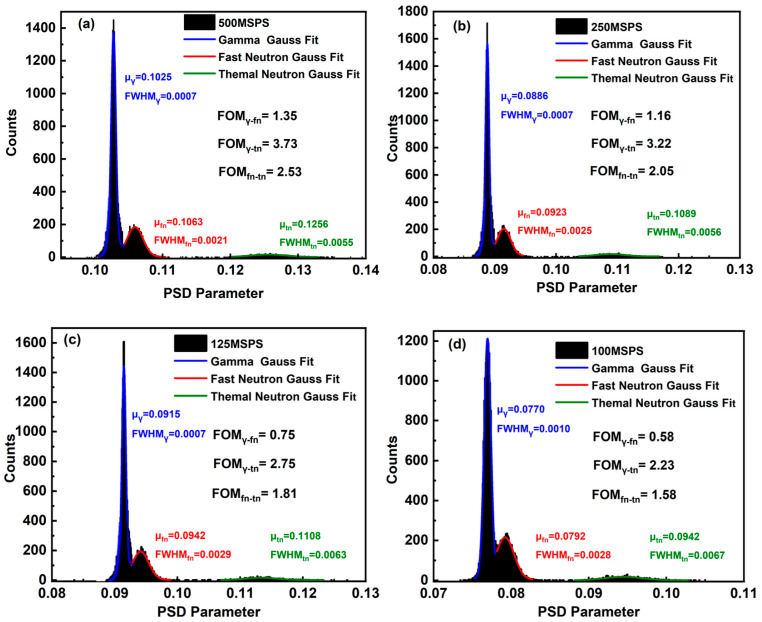
PSP distribution at several different sampling rates. (**a**–**d**) The sampling rates are 500MSPS, 250 MSPS, 125 MSPS, 100 MSPS.

The acquired mixed pulse signals were down-sampled to four sampling rates: 500, 250, 125, and 100 MSPS. After pre-processing involving baseline restoration (using the same method described in Section 3.1: determined by averaging the first ten and last ten sampling points of each pulse signal, with the resulting average value then subtracted from all other sampling points) and pulse amplitude normalization, the optimal integration times for the long and short gates were determined through an iterative search. The results of PSD across these sampling rates is shown in Figure 6, and the corresponding FOM for the three particle types are listed in Table 2.

As shown in Table 2, the FOM values for all three particle types improve considerably with increasing system sampling rate. Among them, thermal neutrons are more readily discriminated against than fast neutrons and γ-rays, which aligns with the earlier analysis. When the sampling rate reaches 250 MSPS or higher, the FOM values for both fast neutrons and γ-rays exceed 1.15, confirming effective discrimination. Conversely, reducing the sampling rate from 250 MSPS to 125 MSPS led to a rapid decrease in their FOM values and a corresponding marked deterioration in discrimination performance—a finding that agrees with the analysis in Refs. [8,27].

Therefore, based on this analysis and considering cost-effectiveness, an ADC with a sampling rate of at least 250 MSPS was selected to digitize the output signals from the composite scintillator detector.

## 4. Circuit Design and Signal Processing

To acquire high-quality digitized signals from the composite scintillator detector, a high-speed ADC acquisition board was designed based on the preceding analysis and literature [8], utilizing the AD9434 chip from Analog Devices. Since the input of AD9434 is differential signal, and the output of detector is single-polarity pulse signal, the AD4937-1 chip is selected to design the differential drive circuit.

**Figure 7:**
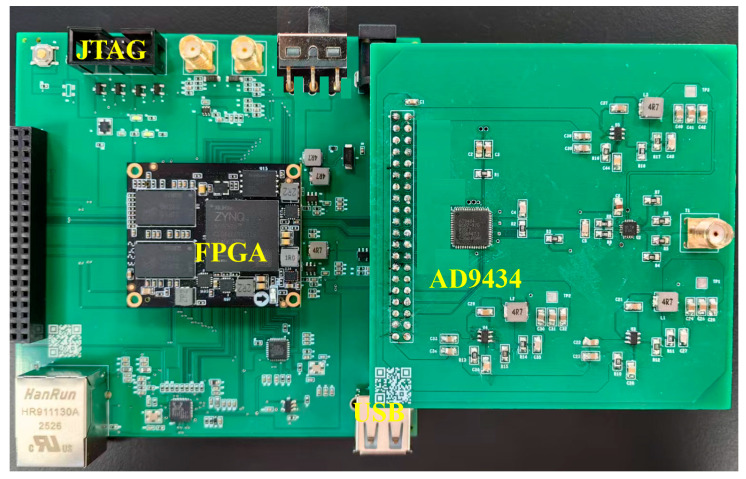
Sampling board with AD9434 and high performance FPGA.

When designing high-speed acquisitong system, the selection of the clock signal is critical for the system to operate stably. Following the AD9434 datasheet recommendations, a clock circuit using the AD9516-1 chip was designed to supply a highly stable, low-jitter clock. Both the clock module and the signal acquisition module are controlled via the SPI bus within the FPGA. The FPGA subsystem was implemented with a ZYNQ XC7Z020 chip. The ADC’s clock, data input, and data output pins were connected to the FPGA baseboard through a 40-pin I/O interface. Interfaces such as USB and Gigabit Ethernet were reserved for data communication, alongside a JTAG interface for program download and configuration. The physical implementation of the ADC acquisition board and FPGA is shown in Figure 7.

After conditioning by the front-end analog circuitry, the detector output signal is routed through an SMA connector to a differential driver. This driver converts the conditioned negative-polarity single-ended pulse signal into a differential pair to meet the ADC’s input requirements for digitization, while simultaneously improving noise immunity, thereby achieving superior digital acquisition performance. The digitized output from the ADC is then transmitted to the FPGA via a high-speed Low-Voltage Differential Signaling (LVDS) interface. PSD logic is implemented within the FPGA fabric. The final computational results, together with the raw waveform data, are transferred to a host computer through a USB 2.0 interface.

**Figure 8:**
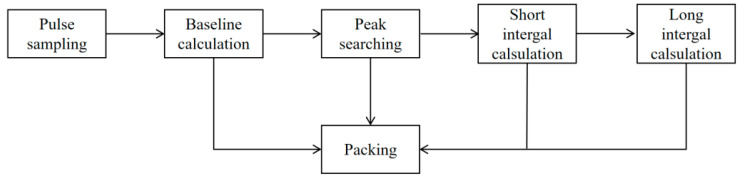
Digital signal processing flow chart.

Data communication between the ADC and the FPGA was accomplished using an LVDS interface, with high-speed acquisition and transmission facilitated by a Xilinx IP core featuring GTX transceivers. Inside the FPGA, the clocking scheme was configured as follows: a differential clock at a specific frequency was supplied to the ADC to establish a 250 MSPS sampling rate, and the trigger mode was set to edge detection for capturing the leading edge of pulse signals. For each acquired pulse, key parameters—including the baseline value, long-gate integral, short-gate integral, and amplitude—were computed. This data was packaged into individual particle data packets and sent to the host computer for subsequent analysis. The complete digital signal processing flow is illustrated in Figure 8.

## 5. Experimental Test and Results

### 5.1. Energy Calibration with γ-Sources

To evaluate the effective neutron detection capability of the system, energy calibration was carried out using standard γ-ray sources (^22^Na, ^137^Cs, and ^60^Co). Each source was placed 5 cm from the detector to ensure relatively uniform irradiation and to minimize pulse pile-up.

**Figure 9:**
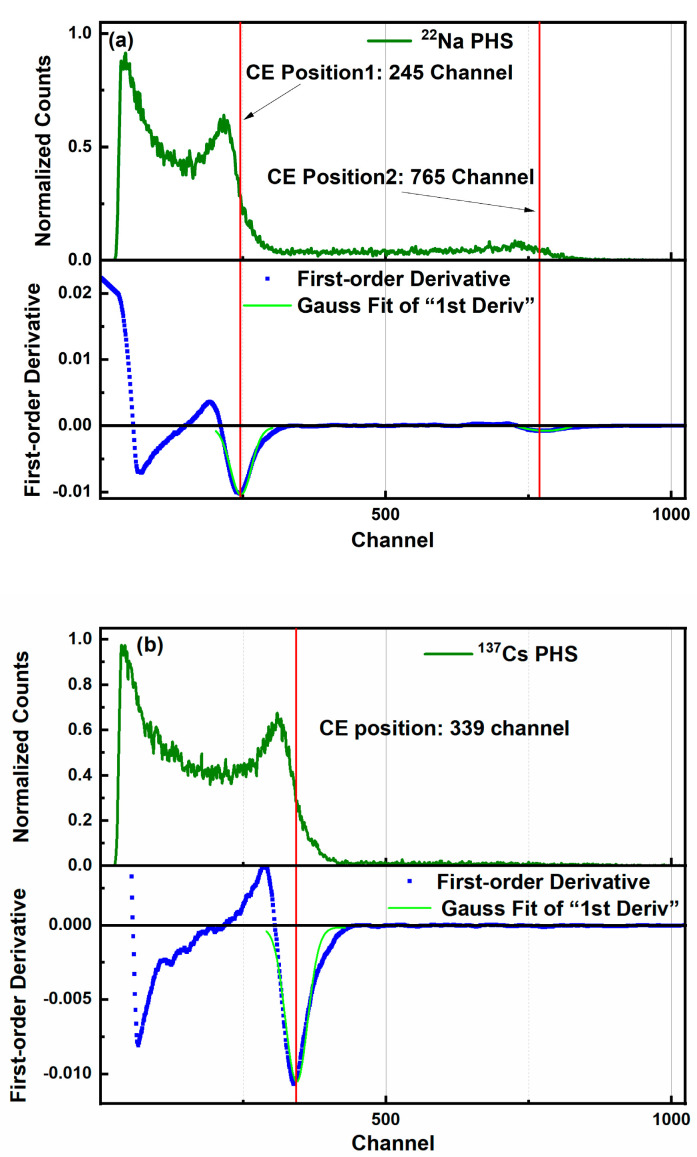

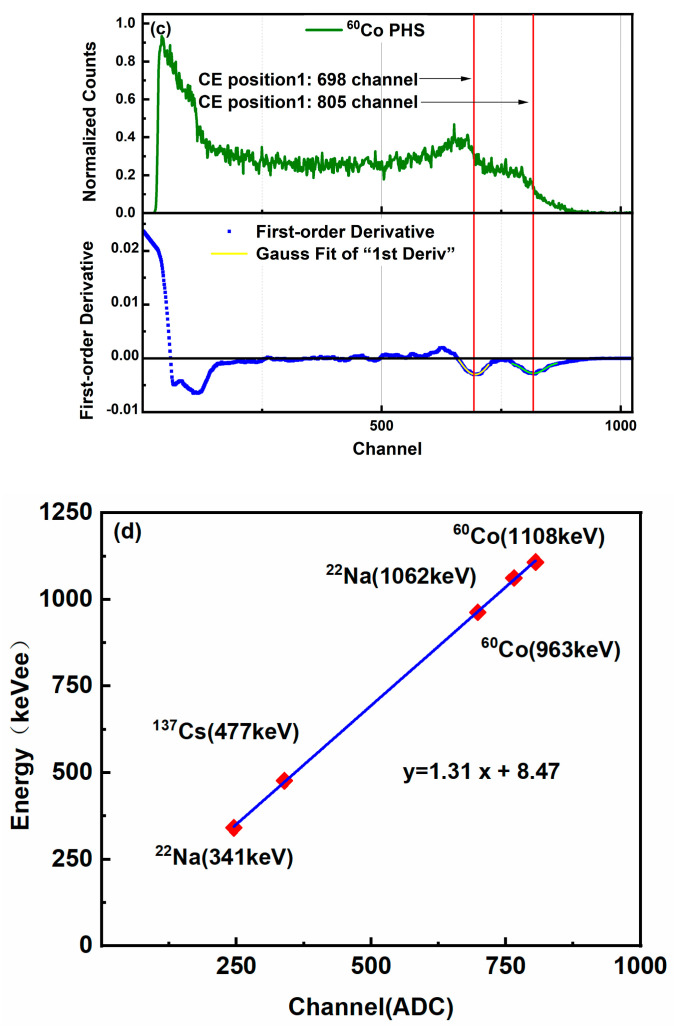
Energy calibration results of gamma rays. (**a**–**c**) Normalized response given by ^22^Na, ^137^Cs, ^60^Co and their first order derivate. (**d**) Energy calibration curves.

The energy spectrum of each source was obtained by integrating the individual pulses. The Compton edge (CE) position was determined by fitting the first-order derivative of the spectrum to an inverse Gaussian distribution, with the centroid of the fit taken as the CE channel, as shown in Figure 9a–c. This approach is consistent with methods used in prior studies [28,29,30]. The calibration curve L (MeVee) was established using a first-order polynomial as follows:

(3) L(MeVee)=a⋅PH(channel)+b

where *a* and *b* are parameters determined by fitting the calibration datapoints. Obtained values are, respectively, *a* = 1.31 and *b* = 8.47, as demonstrated in Figure 9d.

### 5.2. Test Result with n-Source

**Figure 10:**
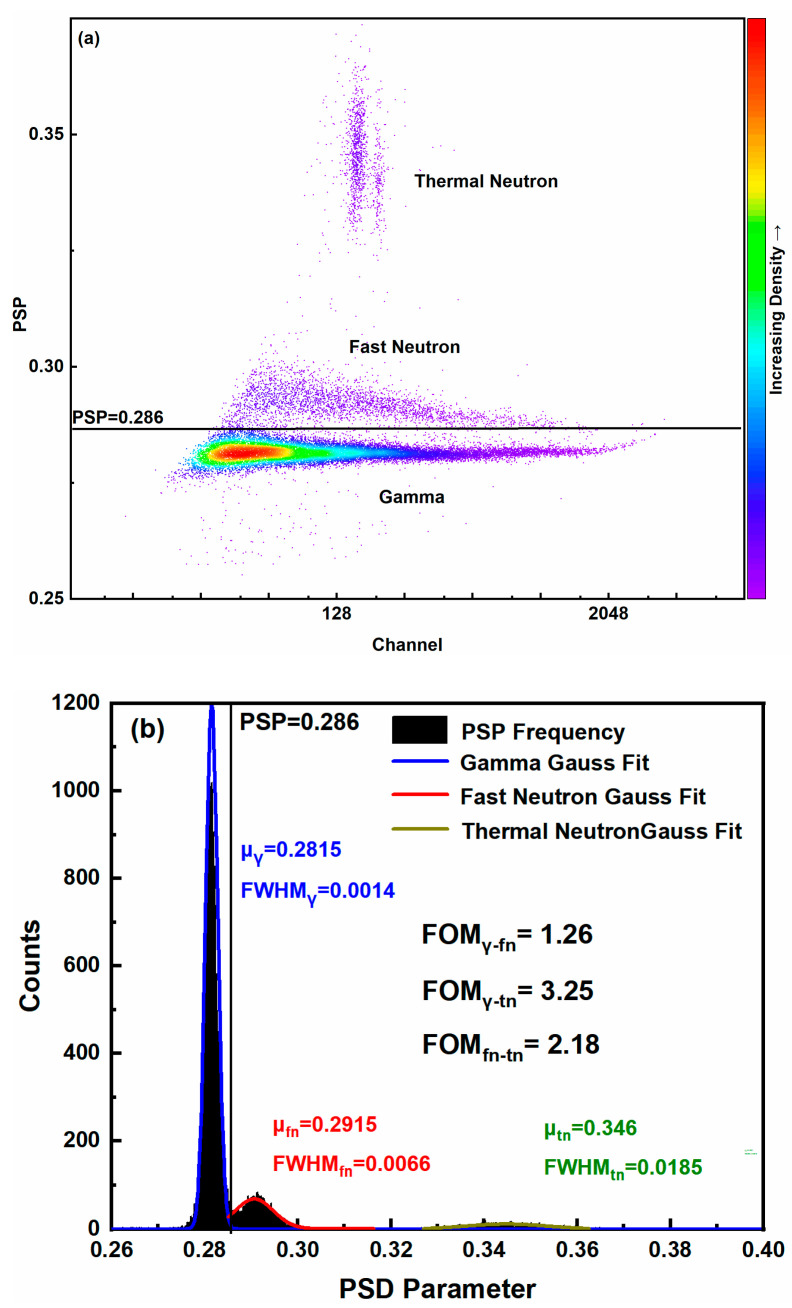
Test results: (**a**) 2D PSD scatter plot for thermal neutron, fast neutron and γ-rays; (**b**) PSP distribution.

Following energy calibration with three γ-ray sources, the discrimination performance of the designed high-speed acquisition board was evaluated in a n–γ mixed field. The discrimination results, derived from the acquisition and processing of a large set of mixed pulse signals, are shown in Figure 10.

As shown in Figure 10, the designed high-speed acquisition board achieved effective triple discrimination. The slight overlap between fast neutron and gamma peak signals in the figure is due to the limited discrimination effect of some highly similar pulse signals at this sampling rate. Based on the noise level, the energy threshold was optimized and set at 50 keV e-equivalent electron energy, the FOM values of fast neutron/γ ray, fast neutron/thermal neutron and γ ray/thermal neutron are 1.26, 2.18 and 3.25, respectively. These results closely match those obtained from the down-sampled DT-5730 digitizer data, confirming that the system meets its design objectives.

### 5.3. False Alarm Rate (FAR)

To comprehensively evaluate the system’s discrimination capability, the false alarm rate (FAR) is defined as the ratio of misidentified events to the total number of events in the test dataset [31]. A discrimination threshold of PSP = 0.286 was applied to classify signals based on their PSP values. The calculated FAR values for the three γ-ray sources are summarized in Table 3. As presented in the table, the system demonstrated FAR values below 0.5% for all three standard γ-ray sources. Although the FAR of ^60^Co is lower than the 0.19% reported in the literature [14], it demonstrates a significant advantage over ^22^Na and ^137^Cs.

### 5.4. Final Discussion-Comparison with Others

Finally, the performance of the proposed design was compared with that of various systems developed by other research teams. Table 4 summarized the key performance parameters of several such systems, all of which employ the CCM for particle discrimination. Under identical conditions, the proposed system demonstrates slightly superior performance to that achieved by down-sampling data originally acquired with the DT-5730 at 500 MSPS and 14-bit resolution. This result indicates that the present design can achieve favorable discrimination performance even at lower sampling rates and resolutions. Comparison with a third system reveals that increasing the sampling rate and resolution significantly enhances discrimination performance between different particles.

Furthermore, the proposed system is unique in its capability for triple discrimination of fast neutrons, thermal neutrons, and γ rays, whereas all other reported systems are limited to discriminating only neutrons and γ rays. It also demonstrates clear advantages over other organic scintillators, as reflected in its superior FOM values.

## 6. Conclusions

This paper presents a high-speed digital triple discrimination system based on a stilbene–^6^Li glass composite scintillator. The system consists of a high-speed acquisition board and an FPGA-based digital processing module, enabling simultaneous discrimination of fast neutrons, thermal neutrons, and γ-rays. To balance discrimination performance and system cost, a down-sampling analysis was conducted on output signals from the composite scintillator detector, which were initially acquired using a DT-5730 digitizer in a ^241^Am–Be neutron field. The analysis shows that the system maintains favorable discrimination performance (FOM value greater than or equal to 1.16) with only minor variations at sampling rates above 250 MSPS, whereas performance degrades rapidly below this threshold. Based on these findings, a digital signal acquisition board was designed with a 250 MSPS sampling rate, a 12-bit ADC, and a high-performance FPGA, capable of real-time waveform discrimination and has the potential for wide-spectrum neutron measurement.

The system was calibrated and evaluated using three γ-ray and neutron sources. Experimental results demonstrate that its discrimination performance for fast neutrons, thermal neutrons, and γ-rays is comparable to that achieved by offline analysis of data from a commercial digitizer. The measured FOM values were 1.26 for fast neutron/γ-ray discrimination, 2.18 for fast/thermal neutron discrimination, and 3.25 for γ-ray/thermal neutron discrimination. Moreover, the FAR was below 0.5% for all three tested γ-ray sources. This system offers a high-performance, cost-effective electronic solution for high-speed acquisition and real-time processing of signals from novel composite scintillator neutron detectors. Future work will integrate time-coincidence measurement capability into the digital system to support neutron spectrum inversion based on the capture gating effect (CGE). Meanwile, the measurement and calculation of the minimum detectable activity (MDA) and minimum detectable neutron flux (MDNF) will be added in the follow-up experiments. This enhancement will enable accurate fast neutron spectrum measurement without host computer control, achieving fully embedded integration of nuclear pulse signal acquisition, processing, and display.

## Figures and Tables

**Table 1:** Properties of stilbene and ^6^Li glass [7,10,19].

Scintillator	Density (g/cm^3^)	Decay Times (ns)	Refractive Index
Stilbene	1.15	4.3, 34.8, 332.0	1.75
^6^Li glass	2.5	16, 59, 290	1.53

**Table 2:** FOM values of the three types of particles at different sampling rates.

Sampling Rates (MSPS)	FOMγ-fn	FOMγ-tn	FOMfn-tn
500	1.35	3.73	2.53
250	1.16	3.22	2.05
125	0.75	2.75	1.81
100	0.58	2.23	1.58

**Table 3:** FAR values obtained with different γ sources.

γ Source	^60^Co	^137^Cs	^22^Na
Total count	67,470	84,057	98,953
False count	220	282	256
FAR (%)	0.33	0.34	0.26

**Table 4:** Comparison of the performance of several different systems [14,26,31].

Detector Composition	Main Performance Parameters of ADC	Identify Particle Species	Equivalent Electron Energy	FOM Value
Stilbene + PMT	250 MSPS/12 bit	fast neutron, thermal neutron, γ-rays	>50 keVee	fn-γ: 1.25, fn-tn: 2.18, γ-tn: 3.25
Stilbene + PMT	250 MSPS/14 bit	fast neutron, thermal neutron, γ-rays	>50 keVee	fn-γ: 1.16, fn-tn: 2.05, γ-tn: 3.22
Stilbene + SiPM	125 MSPS/10 bit	neutron, γ-rays	125 keVee ± 10 keVee	0.95–1.02
BC501A + PMT	1 GSPS/14 bit	neutron, γ-rays	0–4000 keVee	1.005
EJ-309 + PMT	250 MSPS/12 bit	neutron, γ-rays	Not mentioned	1.26

## Data Availability

No new data were created or analyzed in this study. The original contributions presented in this study are included in the article. Further inquiries can be directed to the corresponding author. There was no public involvement in any aspect of this research.
